# Deciphering the genetic architecture of resistance to *Corynespora cassiicola* in soybean (*Glycine max *L.) by integrating genome-wide association mapping and RNA-Seq analysis

**DOI:** 10.3389/fpls.2023.1255763

**Published:** 2023-09-27

**Authors:** Sejal Patel, Jinesh Patel, Kira Bowen, Jenny Koebernick

**Affiliations:** ^1^ Department of Crop, Soil and Environmental Sciences, Auburn University, Auburn, AL, United States; ^2^ Department of Entomology and Plant Pathology, Auburn University, Auburn, AL, United States

**Keywords:** GWAS, target spot, *C. cassiicola*, RNA-Seq, resistance

## Abstract

Target spot caused by *Corynespora cassiicola* is a problematic disease in tropical and subtropical soybean (*Glycine max*) growing regions. Although resistant soybean genotypes have been identified, the genetic mechanisms underlying target spot resistance has not yet been studied. To address this knowledge gap, this is the first genome-wide association study (GWAS) conducted using the SoySNP50K array on a panel of 246 soybean accessions, aiming to unravel the genetic architecture of resistance. The results revealed significant associations of 14 and 33 loci with resistance to LIM01 and SSTA *C. cassiicola* isolates, respectively, with six loci demonstrating consistent associations across both isolates. To identify potential candidate genes within GWAS-identified loci, dynamic transcriptome profiling was conducted through RNA-Seq analysis. The analysis involved comparing gene expression patterns between resistant and susceptible genotypes, utilizing leaf tissue collected at different time points after inoculation. Integrating results of GWAS and RNA-Seq analyses identified 238 differentially expressed genes within a 200 kb region encompassing significant quantitative trait loci (QTLs) for disease severity ratings. These genes were involved in defense response to pathogen, innate immune response, chitinase activity, histone H3-K9 methylation, salicylic acid mediated signaling pathway, kinase activity, and biosynthesis of flavonoid, jasmonic acid, phenylpropanoid, and wax. In addition, when combining results from this study with previous GWAS research, 11 colocalized regions associated with disease resistance were identified for biotic and abiotic stress. This finding provides valuable insight into the genetic resources that can be harnessed for future breeding programs aiming to enhance soybean resistance against target spot and other diseases simultaneously.

## 1 Introduction

Soybean (*Glycine max* L. Merrill) is a principal legume crop that is cultivated in over 50 countries. It provides a valuable source of high-quality oil and protein for human and animal feed. The United States achieved production of approximately 116.3 million metric tons in 2022, representing around 31% of global production (USDA, 2022). However, about 11% of this soybean production is annually constrained by diseases, according to Hartman et al. (2015). The occurrence of target spot, caused by *Corynespora cassiicola*, (Berk & M.A. Curtis) C.T. Wei is a concern for farmers since due to its potential to cause 18 to 32% yield loss (Dixon et al., 2009; Godoy, 2015; Faske, 2016). It was first reported on soybean in 1945 (Olive et al. (1945); it is now considered widespread in the mid-south and the southeast US due to frequent warm and humid conditions that tend to occur during crucial stages of reproductive development.

Disease symptoms initially appear on foliage as lesions with alternating concentric rings of light and dark brown bands encircled by a yellow halo. Severe infections lead to premature senescence of leaves, resulting in yield loss and diminished seed quality (Seaman et al., 1965; Koenning et al., 2006; Godoy, 2015). The foliar pathogen, *C. cassiicola*, belonging to the phylum Ascomycota, has a broad host range, including cotton, tomato, rubber, and cucumber (Blazquez, 1967; Chee, 1988; Barrett et al. 1991; Conner et al., 2013). However, host-specific *C. cassiicola* isolates have also been reported (Dixon et al., 2009; Sumabat et al., 2018).

Current disease control for target spot depends on field management practices and chemical applications. Although fungicides can control target spot, there is a concern over the development of fungicide resistance (Xavier et al., 2013) which may reduce the effectiveness of the fungicide products (Duan et al., 2019; Rondon and Lawrence, 2019). Moreover, fungicidal control of target spot is not always economically or environmentally sustainable. Therefore, development and cultivation of target spot resistant cultivars are needed for economical and enduring strategy for disease management.

Conventional breeding methods for developing resistant soybean cultivars with competitive yield levels involve a lengthy selection process. However, with the availability of abundant molecular markers and affordable genotyping resources, it would be more efficient to identify genomic regions associated with resistance and incorporate them into a marker-assisted breeding program, which would accelerate the selection process and reduce the overall cost of developing new varieties (Neeraja et al., 2007; Hasan et al., 2021).

The soybean cultivar Williams 82 genome was assembled in 2010, spanning 950 Mb distributed in 20 linkage groups (Schmutz et al., 2010). Additionally, reference genomes for two other soybean accessions, ‘Zhonghuang 13’ (ZH13) and wild soybean ‘W05’, are available (Shen et al., 2019; Xie et al., 2019). Six cultivated and two wild relatives of soybean were sequenced and analyzed to develop SoySNP50K, a high-throughput SNP assay to facilitate genotyping (Song et al., 2013). This SNP assay has been used to genotype 18,480 *G. max* accessions and 1168 *G. soja* accessions available in the United States Department of Agriculture (USDA) Soybean Germplasm Collection located at the University of Illinois (Song et al., 2015). The SoySNP50K has played a key role in conducting Genome-wide Association Studies (GWAS) and Quantitative Trait Locus (QTL) studies.

A GWAS associates variation across the whole genome with phenotypes and identifies genetic variations of essential traits. A panel of diverse individuals provide a higher mapping resolution than the classic bi-parental QTL mapping method (Korte and Farlow, 2013). The number of recombination events limit QTL mapping, whereas the association panel has accumulated recombination events over several generations, resulting in improved mapping resolution. In soybean, GWAS studies have successfully identified genes associated with resistance to various pathogens including *Sclerotinia sclerotiorum* (Iquira et al., 2015; Wen et al., 2018), *Pythium sylvaticum* (Lin et al., 2020), *Heterodera glycines* Inchinohe (Vuong et al., 2015; Ravelombola et al., 2020), *Fusarium virguliforme* (Bao et al., 2015; Zhang et al., 2015), *Phytophthora sojae* (Li et al., 2016), *Macrophomina phaseolina* (Coser et al., 2017), and *Cercospora sojina* (McDonald et al., 2023).

Target spot is also a concern in other crops such as rubber, cucumber, tomato, and cotton. Transcriptome profiling studies in cucumber and rubber have elucidated genetic mechanisms underlying resistance to *C. cassiicola* (Wang et al., 2018; Roy et al., 2019; Ribeiro et al., 2021). These studies have identified defense-related genes, biosynthesis of plant hormones, transcription factors, Ca^2^+ signaling pathways, secondary metabolites, and miRNA involved in the defense response of host plants against *C. cassiicola* infection. In rubber, molecular markers linked to tolerance to *C. cassiicola* infection were identified by screening 104 F_1_ clones with 28 SSR markers and performing single marker analysis (Oktavia et al., 2021). Similarly, in cucumber, a combination of SSR and SNP markers were employed to screen a large population of F_2_ plants, leading to the fine mapping of a recessive resistance gene, *cca-3*, against *C. cassiicola* (Wen et al., 2015). Further investigation of candidate genes within the mapped region revealed the presence of the *RGA* (Resistance Gene Analog) gene containing a CC-domain, nucleotide-binding domain (NB-ARC), and the Leucine-rich repeat (LRR) domain, which might play a crucial role in hypersensitive responses to attack by the target spot pathogen (Wen et al., 2015). Valuable genomic resources like reference genomes, a diverse germplasm collection, molecular markers, and other genotyping options are available for soybean; however, *C. cassiicola* resistance has not been investigated at the genomic level. Therefore, this study aims to determine genomic regions associated with resistance against *C. cassiicola* and subsequently identify candidate genes located within identified QTL regions.

## 2 Materials and methods

### 2.1 Plant material and experiment design

A collection of 246 soybean genotypes from different geographic regions (USA, China, Japan, South Korea, North Korea, Vietnam, Nepal, Pakistan, Taiwan, Australia, and Georgia) were obtained from the National Germplasm Database (GRIN). Two genotypes, ‘Council’ (PI 587091) and ‘Henderson’ (PI 665225) were used as resistant and susceptible checks, respectively (Patel et al., 2022). To identify the genomic regions associated with target spot resistance, greenhouse experiments were conducted using two isolates of *C. cassiicola* namely LIM01 and SSTA (Patel et al., 2022) to pinpoint potential candidate genes. These isolates exhibit variations in their toxin producing cassiicolin gene. LIM01 has *Cas2* while SSTA has *Cas2* and *Cas6*. This approach facilitates the identification of horizontal resistance mechanisms. In brief, infected leaf tissue was collected from soybean fields located in Georgia and Alabama. To ensure the isolation of pure cultures, the collected tissue was rinsed with 70% ethanol, 2% NaOCl, and sterile distilled water. The cleaned leaf tissue was placed on Potato dextrose agar plates supplemented with kanamycin to prevent bacterial growth. The plates were incubated at 28°C to obtain fungal colonies. Single colonies from each isolate were transferred to slant tubes containing V8 juice agar. The inoculated slant tubes were incubated at 28°C for two weeks. In order to preserve the isolates for the long term, the slant tubes were kept in a refrigerator set at 4°C. The PCR method was used to confirm the species and pathovar of isolates by utilizing conserved gene sequences.

Soybean genotypes were arranged in a randomized complete block design (RCBD), with three replications. Plants were grown in polypropylene deepots D40L (Stuewe & Sons, Inc., Tangent, OR) filled with potting mix (PRO-MIX BX, Premier Tech Horticulture, Quakertown, PA). Three seeds per deepot were sown and later thinned to one plant per deepot at five days post-emergence. The experiments with LIM01 and SSTA isolates were conducted in Plant Science Research Center, Auburn University, with a temperature range 24°C to 34°C. Halide bulbs (1000-watt) provided supplemental light to ensure a 14-hour photoperiod. Plants were grown to the V3 toV4 stage before inoculation and were watered as needed.

### 2.2 Inoculation and phenotyping

Inoculation and disease assessment methods were followed the procedures described by Patel et al. (2022). Each 10-day old *C. cassiicola* culture, grown on V8 agar, was flooded with sterile distilled water and the colony surface was gently rubbed with a glass rod. The resulting conidial suspension was filtered through double layers of sterile cheesecloth and the concentration was adjusted to 50,000 conidia per ml. All leaves on plants were inoculated by applying the conidial suspension to the upper and lower leaf surfaces using a fine mist professional spray bottle. Subsequently, the inoculated plants were transferred to a mist chamber, with mist running two seconds every 10 minutes over period of 72 hours to create high humidity (>90%). Disease was assessed at 14 days post inoculation based on percentage of damaged leaf area per plant. Following the visual diagram, severity rating scale ranged from 0 to 90%, as outlined by Vincelli and Hershman (2011). Genotypes were categorized as resistant if the disease severity was below 25%, moderately susceptible if the severity was between 25 and 50%, and susceptible if severity exceeded 50%. Descriptive statistics of phenotypic data were performed using PROC GLIMMIX (Generalized linear mixed model) in SAS (version 9.4; SAS Institute Inc., Cary, NC). In this analysis, genotypes, isolates and genotypes×isolates as fixed effect, while replications as random effect.

### 2.3 Genotyping

The 246 soybean accessions were genotyped using Illumina Infinium SoySNP50K BeadChip (Song et al., 2015). This association panel identified 42,180 SNPs. Individual markers with a minor allele frequency <5% or missing rate >10% were omitted from the further analysis. As a result, a high-quality set of 33,378 SNPs was retained for GWAS analysis.

### 2.4 Population structure and linkage disequilibrium estimation

The population structure was analyzed through STRUCTURE v.2.3.4 software to determine subpopulations (Pritchard et al., 2000). The analysis used 33,378 SNPs and employed an admixture model with five iterations of 50,000 burn-in and 50,000 Monte Carlo Markov Chain (MCMC) replications for a range of subpopulations from k=1 to k=10. The optimal number of subpopulations was determined by estimating DeltaK using STRUCTURE HARVESTER (Earl and Vonholdt, 2012). Additionally, linkage disequilibrium (LD) between pairs of SNP markers was evaluated using TASSEL 5 (Bradbury et al., 2007) with a sliding window size of 50 SNPs. The average LD decay rate for all the chromosomes was estimated at the distance the squared correlation coefficient (r^2^) dropped to half its maximum value in this population (Remington et al., 2001; Huang et al., 2010).

### 2.5 Genome-wide association study analysis

The mean scores of disease severity ratings and filtered set of SNPs were used to conduct GWAS analysis using GAPIT v3 software. A statistical model FarmCPU (Fixed and random model Circulating Probability Unification) was employed, with PCA (Principle component analysis) and Kinship as covariates (FarmCPU_(PCA+K) (Liu et al., 2016; Wang and Zhang, 2021). FarmCPU is known to control false positives and false negatives, making a superior model compared to generalized linear model (GLM) and the mixed linear model (MLM), and has been widely used in soybean GWAS studies (Kaler et al., 2018; Chamarthi et al., 2021). The significant threshold for the association between SNPs and target spot severity trait was set at -log10(*P*) ≥ 3.5. The adjusted P value was calculated by False Discovery Rate (FDR) using the Benjamini-Hochberg method.

### 2.6 Prediction of candidate genes

To identify potential candidate genes, a search was conducted within a 200 kb genomic region on both sides of the significantly associated SNPs. The selected candidate genes were then annotated using the Glyma.Wm.82.a2 soybean reference genome available in SoyBase (www.soybase.org) (Cheng et al., 2017; Sun et al., 2020).

### 2.7 Characterization of candidate genes based on RNA-Seq

To identify candidate genes associated with the GWAS-identified loci, we incorporated RNA-Seq data obtained from Patel et al. (2023). Briefly, two resistant genotypes (Bedford and Council) and two susceptible genotypes (Henderson and Pembina) which were inoculated with a *C. cassiicola* isolate at V3-V4 stage and compared to their respective controls (non-inoculated). Total RNA was extracted controls, samples 24 and 48 hours post-infection (hpi) with three biological replications of young leaves using Direct-zol RNA Mini-Prep Kit (Zymo Research, Irvine, CA) according to the manufacturer’s instructions. Subsequently, cDNA library construction and 150-bp paired-end sequencing were carried out by Illumina NovaSeq 6000 instrument (Novo gene Bioinformatics Technology Co., Ltd., CA, US). The raw data were collected, and the low-quality reads, adapters, and Poly A sequences were removed by using fastp software v0.23.1 (Chen et al., 2018). The resulting high-quality reads were aligned to the soybean (*Glycine max* (L.) Merr.) Williams 82 reference genome (Schmutz et al., 2010), and transcript quantification was conducted by Salmon ver.0.9.1 (Patro et al., 2017). To identify genes associated with target spot resistance, R-package DESeq2 (Version 1.37.6) (Love et al., 2014) was applied to determine differential expression analysis. Specifically, genes showing significant changes in expression levels between the *C. cassiicola* inoculated samples, and their respective controls were selected. Moreover, the genes with an absolute log2 fold change ≥ 2 (upregulated genes) or ≤ -2 (downregulated genes) and a false discovery rate (FDR) < 0.01 were considered significant DEGs. The gene ontology (GO) enrichment analysis of differentially expressed genes was performed using Shiny Go v 0.76 (Ge et al., 2020) with *Glycine max* set as the background. The enrichment analysis identified function categories of enriched GO terms, which were considered significant when the false discovery rate (FDR)-adjusted P-value was less than or equal to 0.05.

## 3 Results

### 3.1 Phenotypic analysis

**Figure 1:**
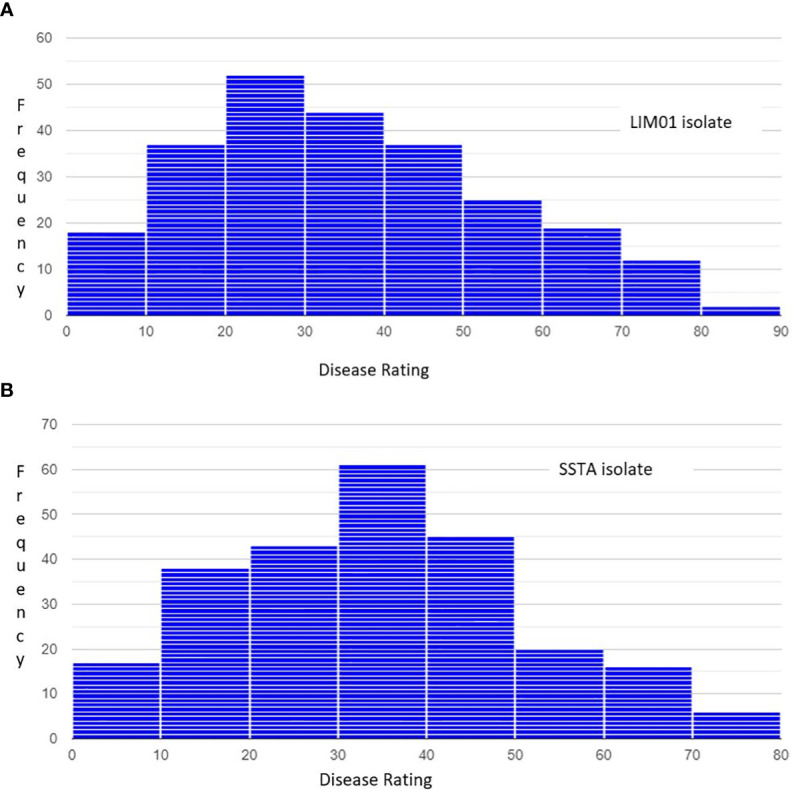
Frequency distribution of disease severity ratings among 246 soybean accessions against *Corynespora cassiicola* infection. Severity ratings for check lines included for reference **(A)** LIM01: The severity ratings for Council is 6.05 and for Henderson is 64.38 **(B)** SSTA: The severity ratings for Council is 5.67 and for Henderson is 61.23.

A total of 246 soybean accessions were evaluated for disease severity to two *C. cassiicola* isolates (LIM01 and SSTA). The phenotypic data obtained from both isolates exhibited a near-normal distribution (**Figure 1**). For isolate LIM01, disease severity scores ranged from 0 to 80%, with an overall mean of 34.2% (**Figure 1A**). Isolate SSTA had disease severity scores ranging from 3.3 to 76.6%, with an overall mean of 32.7% (**Figure 1B**). The disease severity ratings showed a high correlation of (r = 0.85) between the two isolates. Analysis of variance revealed significant differences in genotypes and the genotype by isolate interaction (P <0.0001), while no significant difference was observed between the isolates (P = 0.09) (**Table 1**). Additionally, 77 germplasm lines were identified with less than 25% disease severity when infected by either isolate, providing a wide range of germplasm lines for developing resistant varieties.

**Table 1:** Analysis of variance for soybean accessions with two *Corynespora cassiicola* isolates.

Source of variance	Degree of freedom	F value	*P* Value
Genotype	245	63.08	<0.0001
Isolate	1	9.8	0.0887
Genotype × Isolate	245	3.19	<0.0001

### 3.2 Distribution of SNP markers and linkage disequilibrium decay

**Figure 2:**
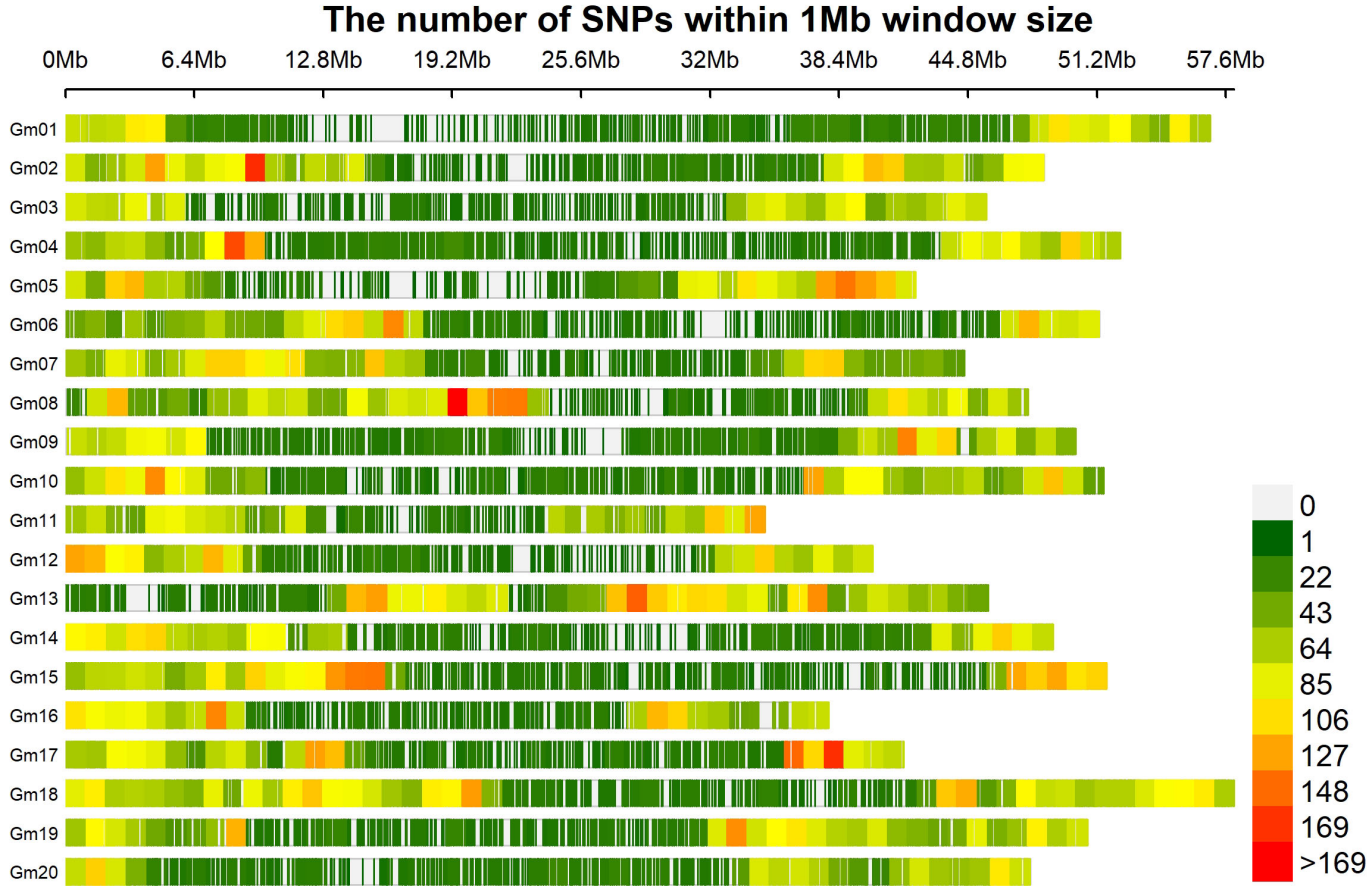
Distribution of SNP markers across the soybean genome. Different color represent the SNPs marker contained within 1Mb region. The red area indicates SNPs rich region, and the white area in between indicates the absence of SNP markers.

The 33,378 SNPs were distributed across all twenty chromosomes (**Figure 2**), with an average of 1669 SNPs per chromosome. Chromosome 18 exhibited the highest number of SNPs (2771 SNPs), while chromosome 20 had the lowest number (1106 SNPs). The average distance between the two markers was approximately 28 kb. Chromosome 18 had the smallest inter-marker distance of 21 kb, while chromosome 20 had the largest distance of 43.3 kb.

**Figure 3:**
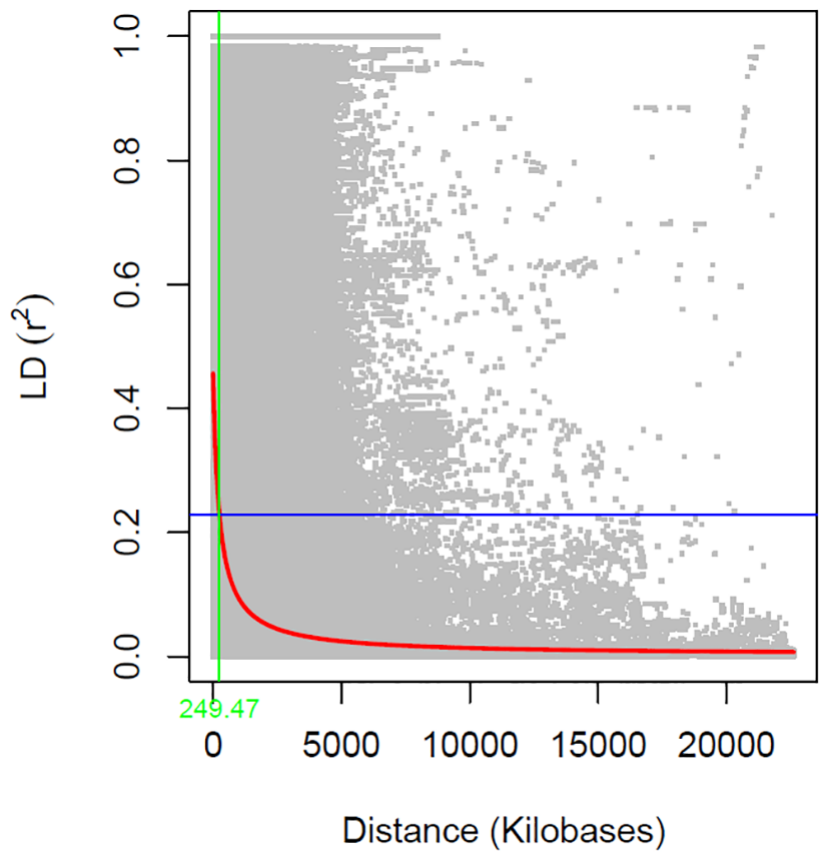
Genome-wide linkage disequilibrium (LD) decay rate estimated in 246 soybean accessions. The X- axis represents the genetic distance in Kb (killo bases) and Y-axis represents the squared correlation coefficient (r^2^).

The linkage disequilibrium (LD) analysis filtered 33,378 SNPs to calculate pairwise r^2^ value, which were plotted against the physical distance. The fitted curve (red line) in the graph estimated the rate of LD decay (**Figure 3**). The blue lines represented half the maximum r^2^ value, and the intersection of the green and blue lines indicated the distance in kilobase pair (kb) the maximum r^2^ value dropped to half. The estimated LD decay rate reached half of its maximum value at approximately 249.47 kb, which is consistent with previous soybean GWAS studies (Hwang et al., 2014; Dhanapal et al., 2015).

### 3.3 Population structure

**Figure 4:**
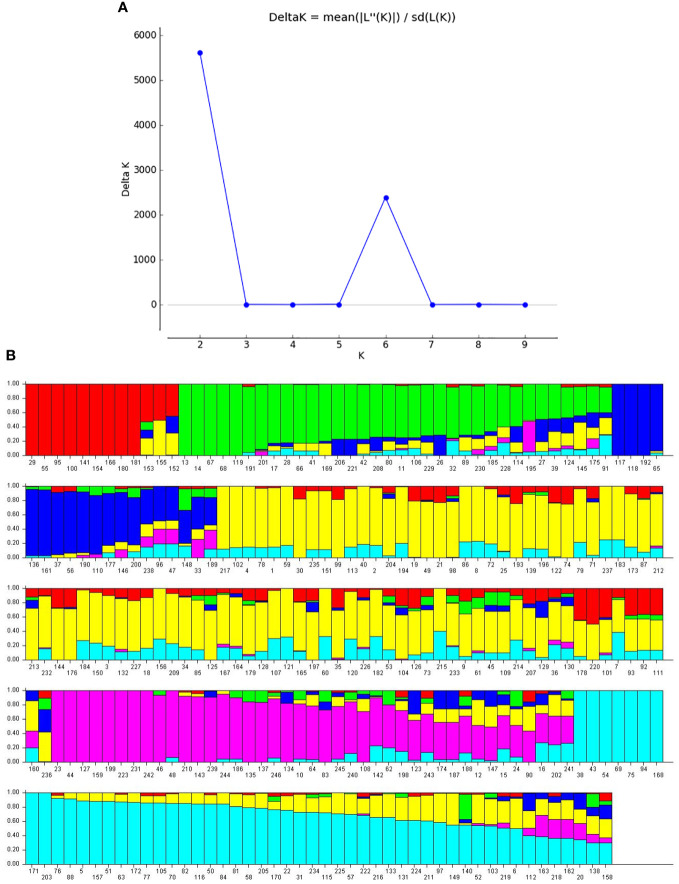
Population structure of the 246 soybean accessions. **(A)** Plot of delta k against putative k from 1 to 10. **(B)** Population structure estimates (k=6). Numbers on the X-axis represent germplasm and the membership coefficient displayed on the Y-axis. Accessions sharing the same group are assigned the same color.

Population structure analysis was performed using STRUCTURE software to determine the number of subpopulations among 246 genotypes. The likelihood value [LnP(D)] was obtained from analysis using the 33,378 SNPs was used to calculate the delta K value, which determined the best-fitted subpopulation. The delta K graph showed two peak values at K=2 and K=6. However, the best-fitted subpopulation group was determined as K=6 because it successfully differentiated soybean accessions collected from different locations (**Figure 4A**). Under K=6, the 246 accessions were distributed across six groups: group 1 (12 accessions), group 2 (34), group 3 (19), group 4 (87), group 5 (41), and group 6 (53) (**Figure 4B**). Further, groups 1 and 4 primarily comprised accessions from South Korea. At the same time, China and Vietnam dominated group 2. Accessions from China were predominantly present in group 3, accessions from the US were mainly found in group 5, and accessions from Japan prevailed in group 6 (**Supplementary Table 1**).

### 3.4 Genomic regions identified for target spot

**Figure 5:**
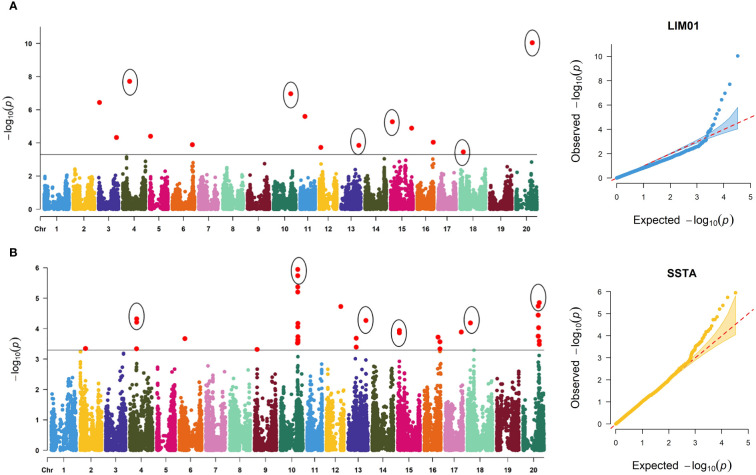
Manhattan and quantile-quantile (QQ) plots displaying significantly associated SNPs for target spot against two *C. cassiicola* isolates: **(A)** LIM01 and **(B)** SSTA. The cut-off threshold is −log10 (*p*) ≥ 3, represented by a black horizontal line.

A total of 14 marker-trait associations with a significance threshold of −log10 (P) ≥ 3.5 and P ≤ 0.0003 were identified with disease severity ratings in response to the LIM01 isolate. These associations were found across 12 chromosomes of the soybean genome, with chromosomes 3 and 15 harboring two associated SNPs each (**Table 2**, **Figure 5A**). Notably, the allelic effect for disease severity ranged from -7.92 (ss715584729, Chr. 3) to 6.5 (ss715637816, Chr. 20). In terms of traits involving disease ratings, a major allele is desirable when the allele effect is positive, whereas a minor allele is preferred for the disease resistance when allele effect is negative.

**Table 2:** The SNPs (single-nucleotide polymorphisms) associated with target spot resistance with each of two *C. cassiicola* isolates.

Isolate	SNP Marker	Chr^a^	Position (bp)	−log10 (*P*-Value)	FDR^b^	Allelic effect^c^	MAF^d^
LIM01	ss715584729	03	2,091,140	6.436	0.003056	-7.915	0.132
	ss715586174	03	40,847,194	4.321	0.176922	-6.19	0.108
	ss715587169	04	15,841,584	7.713	0.000323	5.72	0.246
	ss715590348	05	1,952,710	4.4	0.166151	-4.012	0.421
	ss715594534	06	46,295,298	3.885	0.395674	-3.575	0.423
	ss715606800	10	39,534,982	6.964	0.00121	4.825	0.329
	ss715609438	11	11,087,536	5.593	0.017023	-4.842	0.248
	ss715612322	12	3,373,730	3.725	0.483678	3.445	0.262
	ss715616244	13	40,878,186	3.845	0.397328	4.061	0.272
	ss715621861	15	4,137,385	5.273	0.029659	-5.218	0.236
	ss715622335	15	48,361,099	4.884	0.062293	-4.857	0.173
	ss715624869	16	36,571,566	4.035	0.30799	-3.614	0.374
	ss715632608	18	7,161,394	3.449	0.847014	5.94	0.057
	ss715637816	20	37,162,511	10.039	3.05E-06	6.454	0.264
SSTA	ss715587167	04	15,781,556	4.322	0.173075	-5.389	0.423
	ss715587169	04	15,841,584	4.207	0.173075	5.502	0.246
	ss715592934	06	12,584,572	3.672	0.30788	5.686	0.167
	ss715606800	10	39,534,982	5.943	0.030607	6.255	0.329
	ss715606796	10	39,521,125	5.737	0.030607	6.258	0.301
	ss715606801	10	39,543,852	5.371	0.047377	5.847	0.333
	ss715606793	10	39,500,426	5.205	0.052058	6.288	0.266
	ss715606833	10	39,716,038	4.171	0.173075	5.775	0.248
	ss715606779	10	39,456,010	4.055	0.208483	5.411	0.289
	ss715606822	10	39,670,594	3.734	0.301446	-5.779	0.189
	ss715606823	10	39,682,612	3.636	0.30788	4.904	0.254
	ss715606832	10	39,714,825	3.61	0.30788	4.695	0.321
	ss715606829	10	39,709,662	3.562	0.30788	-5.948	0.167
	ss715606817	10	39,666,999	3.547	0.30788	4.861	0.252
	ss715606824	10	39,687,502	3.544	0.30788	5.279	0.323
	ss715606653	10	38,753,114	3.528	0.309125	-5.303	0.222
	ss715612259	12	33,175,894	4.723	0.090209	-8.969	0.091
	ss715616244	13	40,878,186	4.268	0.173075	6.35	0.272
	ss715615362	13	18,693,392	3.686	0.30788	-6.957	0.14
	ss715621861	15	4,137,385	3.937	0.241394	-6.513	0.236
	ss715621851	15	4,119,316	3.867	0.2519	6.639	0.23
	ss715624393	16	31,349,749	3.722	0.301446	-5.101	0.35
	ss715624785	16	35,789,070	3.568	0.30788	5.876	0.283
	ss715627202	17	37,097,907	3.889	0.2519	-5.52	0.346
	ss715632608	18	7,161,394	4.185	0.173075	9.985	0.057
	ss715637816	20	37,162,511	4.85	0.090209	5.846	0.264
	ss715637481	20	34,776,314	4.738	0.090209	-5.631	0.419
	ss715637482	20	34,795,864	4.444	0.150129	-5.463	0.415
	ss715637615	20	35,651,832	4.028	0.208483	-4.514	0.498
	ss715637471	20	34,699,114	3.755	0.301446	5.219	0.396
	ss715637820	20	37,173,335	3.594	0.30788	4.947	0.335
	ss715637817	20	37,167,430	3.591	0.30788	4.95	0.327

a Chromosome.

b False discovery rate (FDR) adjusted p values.

c Minor allele effect of the SNP.

d Minor allele frequency.

For disease severity ratings in response to the SSTA isolate, a total of 33 marker-trait associations were discovered (**Figure 5B**). These associations were distributed among 10 chromosomes, with chromosome 10 exhibiting the highest number of SNPs (13) and chromosome 20 having seven associated SNPs. Additionally, the allelic effect for disease severity ranged from -8.96 (ss715612259, Chr. 12) to 9.98 (ss715632608, Chr. 18).

Common significantly associated SNPs for disease severity ratings for both *C. cassiicola* isolates are important to develop a marker-based selection strategy for target spot. Six SNPs were found to be significantly associated with disease severity ratings for both *C. cassiicola* isolates (**Figure 5**). Among these SNPs, only one of the SNP (ss715621861 on Chr. 15) exhibited a negative allelic effect, while the remaining five common SNPs showed a positive allele effect for disease severity ratings in response to both isolates (**Table 2**). Among the 77 lines identified with disease severity below 25% upon infection with either isolate, the majority had favorable alleles for ss715587169 (83.1%), ss715606800 (79.2%), and ss715632608 (98.7%). However, only 39%, 55.8%, and 57.1% had favorable alleles for ss715621861, ss715637816, and ss715616244 respectively.

### 3.5 Candidate genes associated with target spot resistance

A total of 41 SNPs had significant associations with severity ratings in response to the two isolates. Candidate genes were searched for in the vicinity of 200kb region on either side of the associated SNPs. Several markers were identified on chromosomes 10 and 20 associated with disease severity to SSTA isolate; instead of gene mining for all the SNPs on these chromosomes, only a few markers were selected that can span the entire region. Three markers were selected to cover the 13 SNPs in the region of chromosome 10, while two markers were chosen to cover the seven SNPs in the region of chromosome 20. In total, 615 and 640 genes were found in the vicinity of associated SNPs for disease ratings in response to LIM01 and SSTA isolates, respectively (**Supplementary Table 2**). Additionally, the six SNPs associated with disease severity ratings for both isolates were found to have 262 genes. Functional annotation of these genes revealed their involvement in many important defense pathways, such as against fungi, and other organisms such as bacteria, viruses, and oomycetes. In addition, chitin binding and chitinase activity, MAPKKK cascade, protein serine/threonine kinase activity, response to ethylene, biosynthesis of lignin, flavonoid, salicylic acid, brassinosteroid, and jasmonic acid (Grant et al., 2010; Morales et al., 2013).

### 3.6 Integration of information from GWAS and RNA-Seq analysis

**Figure 6:**
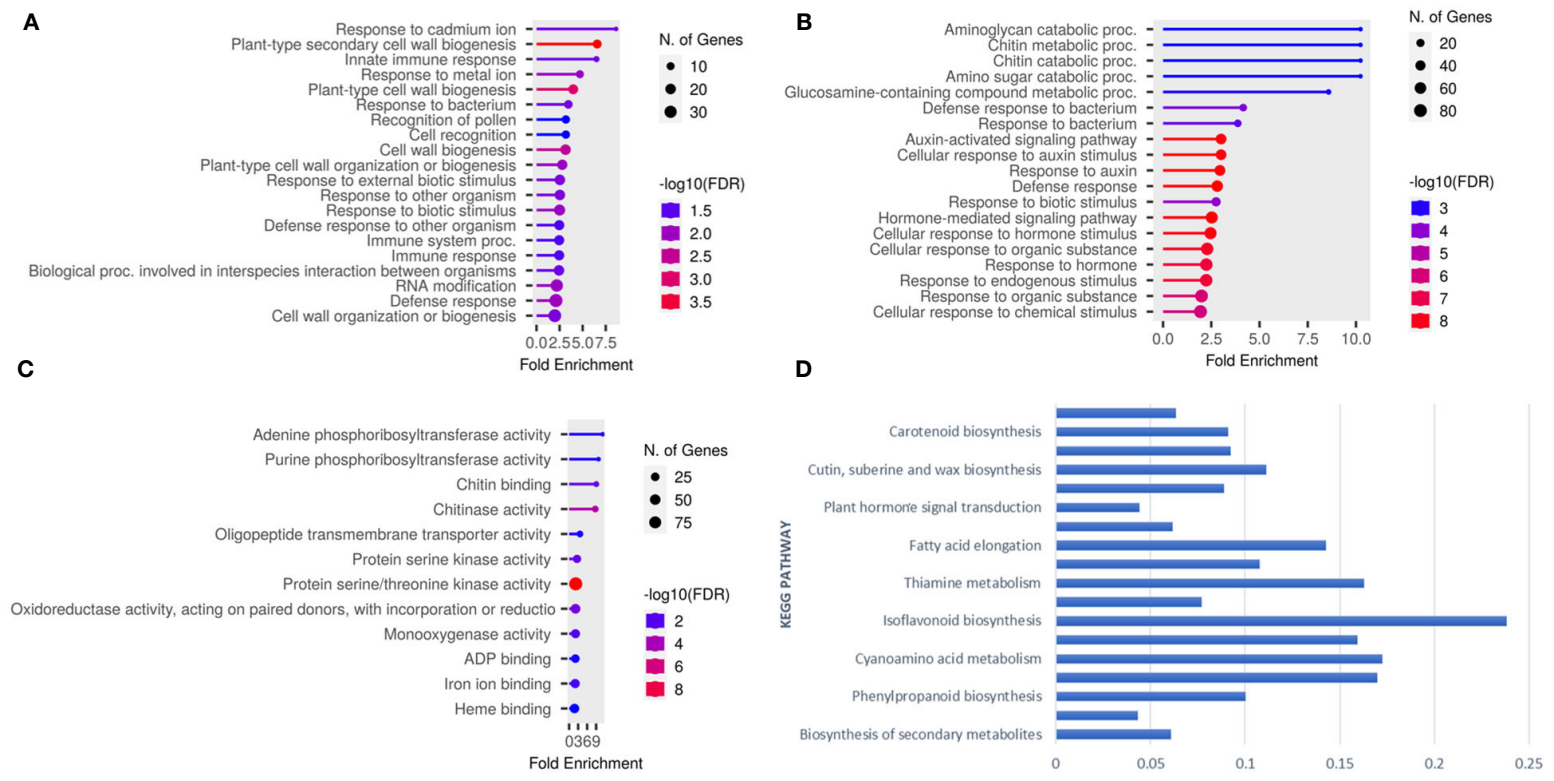
Go term analysis conducted on upregulated genes within resistance genotypes yielded plots illustrating: **(A)** Biological processes for Bedford **(B)** Biological processes for Council, **(C)** Molecular processes for Council, and **(D)** Represent significantly enriched KEGG pathways identified for differentially expressed genes (DEGs) that were shared across all four genotypes.

To gain further insights into the genetic basis of target spot resistance, a transcriptomic study was conducted for two resistant (Council and Bedford) and two susceptible (Henderson and Pembina) genotypes at different timepoints during *C. cassiicola* following inoculation. RNA-Seq analysis identified differential gene expression patterns, with 6480 being upregulated and 7890 genes being downregulated in at least one of these genotypes (**Supplementary Figure 1**). The GO and KEGG analyses uncovered the response of the resistant and susceptible genotypes to infection by *C. cassiicola*. GO analysis of upregulated genes in Council enriched the biological and molecular functions process, while Bedford showed that biological processes were enriched. The Bedford genotype showed enriched biological processes for defense response, response to external biotic stimulus and immune responses, and plant-type cell wall biogenesis (**Figure 6A**). The upregulated DEGs in Council enriched processes like defense response, hormone-mediated signaling, chitin metabolism/catabolism, and responses to auxin and biotic stimuli (**Figure 6B**). Regarding molecular function, protein serine/threonine kinases, chitin binding, chitinase, and oxidoreductases. Certain molecular functions, such as protein serine/threonine kinase activity, chitinase activity, and oxidoreductase activity, were also increased (**Figure 6C**). Furthermore, the KEGG enrichment analysis was applied to the upregulated DEGs that were commonly observed across all four genotypes after being inoculated in response to *C. cassiicola*. The results revealed significant enrichments (with FDR-adjusted p ≤ 0.05) in several key pathways (**Figure 6D**). These pathways include metabolic pathways, biosynthesis of secondary metabolites, phenylpropanoid biosynthesis, flavonoid biosynthesis, plant hormone signal transduction, and MAPK signaling pathway.

To integrate the RNA-Seq differentially expressed genes (DEGs) with the candidate genes identified through GWAS analysis, a comprehensive analysis was performed. A total of 131genes in the vicinity of associated markers were upregulated after *C. cassiicola* inoculation in at least one of the genotypes. Among these genes, 24 were specifically upregulated only in the resistant genotypes, while 31 genes were upregulated only in the susceptible genotypes. Furthermore, 115 genes located near the associated markers showed downregulation after *C. cassiicola* inoculation, with 29 genes being specifically downregulated only in the resistant genotypes and 16 genes downregulated only in the susceptible genotypes (**Supplementary Table 3**). Notably, eight genes exhibited interesting expression patterns after *C. cassiicola* inoculation, showing upregulation in one genotype while being downregulated in another genotype (**Supplementary Table 3**). These genes may be involved in complex regulatory mechanisms underlying target spot resistance and warrant further investigation.

## 4 Discussion

Target spot is a significant threat to soybean production, particularly in warm and humid regions, and potentially leading to substantial economic losses. With the availability of a well-assembled genome sequence, extensive marker information, and a diverse germplasm collection in soybean (Song et al., 2013), it is possible to explore the relationship between phenotypic variation and genetic information. To the best of our knowledge, this study represents the first attempt to identify SNP markers associated with target spot resistance in soybean.

Disease severity ratings varied greatly among genotypes for each isolate, providing valuable data along with genotypic information for identifying marker-trait associations. In this study, 14 and 33 marker-trait associations were identified for the LIM01 and SSTA isolates of *C. cassiicola*, respectively. By examining the candidate genes surrounding the associated SNPs, a total of 993 candidate genes were discovered. To narrow down this list and enhance confidence in identifying target spot defense-associated genes, the GWAS results were scrutinized and combined with RNA-Seq data of soybean gene expression after *C. cassiicola* infection. This integration led to the identification of 238 differentially expressed genes (DEGs) among the candidate genes. Among the 238 genes, several were determined to have a role in the defense response to pathogen attack. These genes were involved in defense response to pathogen, innate immune response, chitinase activity, histone H3-K9 methylation, salicylic acid mediated signaling pathway, kinase activity, and biosynthesis of flavonoid, jasmonic acid, phenylpropanoid, and wax.

Leucine-rich repeat (LRR) domains are commonly found in plant immune receptors and defense genes. LRR-receptor-like kinase (RLK) genes are primarily involved in development and stress responses and play a crucial role in plant defense mechanisms. Flagellin-sensitive 2 (FLS2), a LRR-RLK gene binds bacterial flagellin and initiates pathogen-associated molecular patterns (PAMPs)-triggered immunity (Chinchilla et al., 2007). LRR-RLK genes have also been implicated in resistance to various pathogens, such as *Cercospora sojina* (McDonald et al., 2023) and *Phytophthora sojae* (Wang et al., 2021) in soybean, *Blumeria graminis* in wheat (Hu et al., 2018), *Hyaloperonospora arabidopsidis* in Arabidopsis (Hok et al., 2011) and *Fusarium graminearum* in barley and wheat (Thapa et al., 2018). The RPS2 gene belongs to a LRR Class of disease resistance that plays a vital role in conferring resistance to a broad spectrum of pathogens (Li et al., 2019). In our study, four LRR-RLK genes and four genes with LRR domains belonging to the disease resistance family were differentially expressed and located near associated SNPs, suggesting their potential involvement in target spot resistance.

RPM1-interacting protein 4 (RIN4) was found near ss715621861 on Chromosome 15, was differentially expressed after *C. cassicola* infection. RIN4 is a plant immunity regulator that plays a crucial role in both PAMP-triggered immunity (PTI) and effector-triggered immunity (ETI) (Zhao et al., 2021). Notably, RIN4 is an inherently disordered protein (IDP) except for the region involved in pathogen-induced post-translational modifications. Functionally, it acts as a central hub protein, binding to multiple proteins and serving as a center for immune signaling pathway formation (Cui et al., 2010; Liu et al., 2011; Oldfield and Dunker, 2014; Toruño et al., 2018).

Plant-specific NAC domain proteins are the class of transcription factors that play crucial role in regulating stress tolerance and defense response against various abiotic and biotic challenges (Oh et al., 2005; Puranik et al., 2012; Yuan et al., 2019). In the narrowed-down candidate genes list, four genes were identified: NAC1, NAC47, and two copies of NAC90. NAC1 gene has been shown to trigger a defense signal upon infection of *Pseudomonas syringae pv*. *tomato*in tomato (Kud, 2017). NAC090, along with other NAC transcription factors, plays an essential role in regulating accumulation of salicylic acid and leaf senescence (Kim et al., 2018; Zhang et al., 2020). In cotton, both NAC1 and NAC90 are involved in stress response to the fungal pathogen *Verticillium dahlia* and positively regulate resistance to verticillium wilt (Wang et al., 2016; Bai et al., 2022).

The GWAS analysis identified two copies of the plant U-box protein PUB23 and one copy of PUB25, were differentially expressed after *C. cassiicola* infection in RNA-Seq analysis. PUB23, along with other U-box proteins, has been found to negatively affect tolerance to drought and *Fusarium oxysporum* (Cho et al., 2008; Chen et al., 2014). Similarly, PUB25, along with PUB26, plays a role in the degradation of MYB6 and negatively impacts resistance to *Verticillium dahlia* (Ma et al., 2021). Exploring the structural and functional characteristics of these U-box proteins and their specific impact on *C. cassiicola* resistance would provide valuable insights into the underlying molecular mechanisms.

Durable resistance against multiple pathogens can be achieved through the manipulation of genes encoding a small number of pathogen recognition proteins. It has been observed that several genes and pathways play common roles in response to both abiotic stress and pathogen attacks. For example, the phenylpropanoid pathway is activated in response to pathogen attack as well as iron deficiency chlorosis (IDC) (Zabala et al., 2006; Waters et al., 2018). The RLK, *GmRLK18-1* has been found to confer pleiotropic resistance to both *Fusarium virguliforme* and *Heterodera glycines* (Srour et al., 2012). Similarly, a set of two genes (*GmSNAP18* and *GmSNAP11*), has been shown to provide resistance against both *Heterodera glycines* and *Rotylenchulus reniformis* (Usovsky et al., 2021). A close linkage of favorable alleles and the pleiotropic effect of genes play an essential role in marker-based selection process, enabling the simultaneous improvement of multiple traits. This approach is particularly beneficial for traits controlled by a few genes, such as disease resistance.

By collating the genomic regions associated from the current study and the previous publications assisted in identifying 11 colocalized regions in the genome linked to different biotic and abiotic stress, such as soybean cyst nematode (SCN), reniform nematode (RN), stem and root rot (*Phytophthora sojae)*, sclerotinia stem rot (*Sclerotinia sclerotiorum*), sudden death syndrome (*Fusarium virguliforme*), yellow mosaic virus, and IDC (**Supplementary Table 4**).

One notable region, a block of less than two Mb on Chr.10 which contains 13 marker-trait association (MTA) for target spot, 14 for *Phytophthora sojae*, and one each for RN and SCN. This region encompasses several genes involved in defense mechanisms such as pathogenesis-related 4, LRR-RLK, *Arabidopsis* toxicos en levadura 6 (ATL6), enhanced disease resistance 1 (EDR1), WRKY3, WRKY4, MYB15, pentatricopeptide repeat, and genes belonging to protein kinase family. Pathogenesis-related protein (PR) are encoded by host genes that are selectively activated under pathogen or parasitic attack to provide a long-term resistance. These genes play a pivotal role in systemic acquired resistance in uninfected tissue distal to the original infection site (Sudisha et al., 2012; Jain and Khurana, 2018). Transcription factors like WRKY3, WRKY4 and MYB15 are prominent families involved in large-scale transcriptional reprogramming during plant immune responses against pathogen attacks (Eulgem and Somssich, 2007; Bosamia et al., 2020). For example, *WRKY3* and *WRKY4* has been shown to enhance plant resistance to necrotrophic pathogens such as *Botrytis cinerea* in *Arabidopsis* (Lai et al., 2008). MYB15 activates lignin biosynthesis genes as part of effector-triggered immune responses, providing resistance to *Pseudomonas syringae* (Kim et al., 2020). In RNA-Seq study, some of these genes showed distinctive expression patterns in the four genotypes upon *C. cassiicola* inoculation (**Supplementary Figure 2A**).

Another region of interest is a 1.86 Mb block on Chr. 16, which contains two MTAs for target spot, one each for *Sclerotinia sclerotiorum*, IDC, and SCN. A heat map displaying DEGs identified within the 1.86 Mb block. It showcases the gene expression changes when comparing 24 hpi and 48 hpi of *C. cassiicola* with control for all four genotypes (**Supplementary Figure 2B**). This region contains 17 gene copies of disease resistance family protein/LRR family protein, four copies of disease resistance protein belonging to TIR-NBS-LRR family, four copies of receptor like protein (RLP), three copies of cysteine-rich RLK protein kinase 25, three copies of cytochrome P450 family protein, two copies of LRR-RLK, and two copies of LRR transmembrane protein kinase. These findings suggest the presence of closely linked blocks that confer resistance to various biotic and abiotic stress. The identification of these regions provides valuable insights for developing marker-assisted selection strategies in soybean breeding programs, enabling the development of cultivars with broad-spectrum resistance against multiple pathogens.

## 5 Conclusion

This study represents the first GWAS conducted in soybean to elucidate the genetic basis of resistance to target spot, a fungal-incited disease. The GWAS analysis identified six common SNPs associated with disease traits in response to two different *C. cassiicola* isolates. Furthermore, the integration of GWAS and RNA-Seq data allowed for the identification of 238 DEGs, several of which were found to be involved in LRR-RLK, LRR protein belonging to disease resistance disease resistance family protein, NAC transcription factor, cytochrome P450, RIN4, Pentatricopeptide repeat (PPR) protein, and plant U-box protein. Moreover, the study revealed the presence of 11 colocalized regions distributed across different chromosomes, which were found to contribute to multiple disease resistances. These regions represent potential QTL harboring genes associated with resistance against various biotic stresses. The identified genetic markers and candidate genes can serve as valuable resources for developing marker-assisted selection strategies, facilitating the development of improved soybean cultivars with enhanced resistance to target spot and potentially other related diseases.

## Data availability statement

The data presented in the study are deposited in the National Center for Biotechnology Information (NCBI) repository, accession number is PRJNA1015685. Illumina Infinium SoySNP50K Bead Chip data is available in the Soybase database (https://www.soybase.org).

## Author contributions

SP: Conceptualization, Data curation, Formal Analysis, Methodology, Visualization, Writing – original draft, Writing – review & editing, Investigation, Software. JP: Conceptualization, Formal Analysis, Methodology, Software, Writing – review & editing. KB: Methodology, Writing – review & editing. JK: Conceptualization, Funding acquisition, Methodology, Resources, Supervision, Writing – review & editing.
