# Supplementary material for: Deciphering the genetic architecture of resistance to Corynespora cassiicola in soybean (Glycine max L.) by integrating genome-wide association mapping and RNA-Seq analysis
Source: Front Plant Sci. 2023 Sep 27;14:1255763. doi: 10.3389/fpls.2023.1255763 (PMC10565807; doi:10.3389/fpls.2023.1255763)
Supplement: Supplementary file 1 [file DataSheet_1.zip › Supplementary_materials_1255763_manuscript_9_15_2023/Updated_Supplementary_figure_1255763_manuscript.docx]

B

A

| 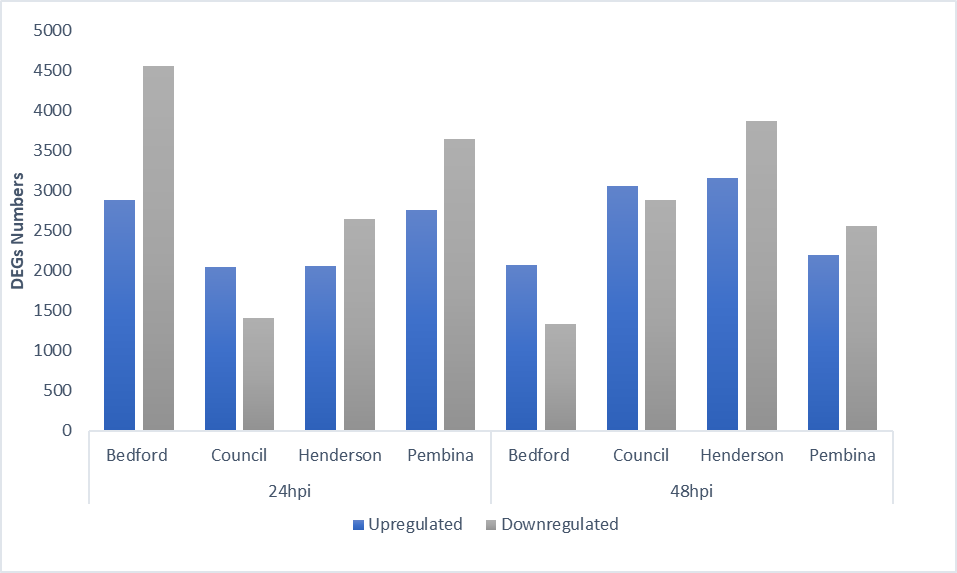 | |
| --- | --- |
| 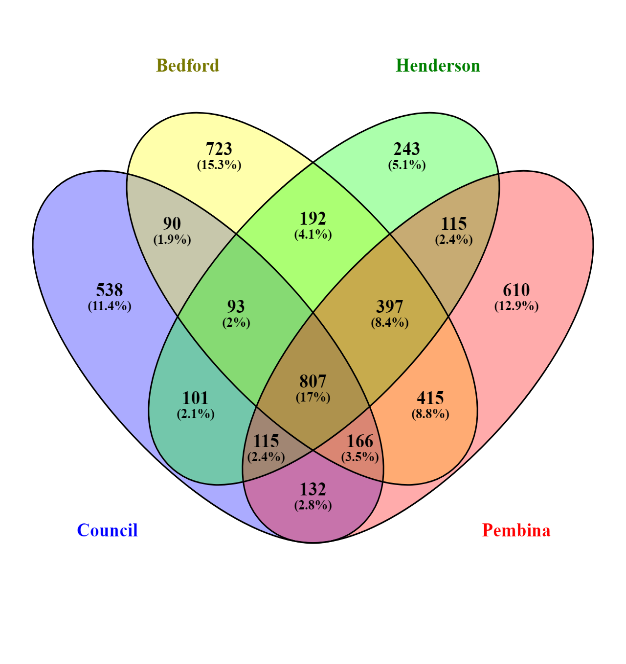 24hpi upregulated genes | 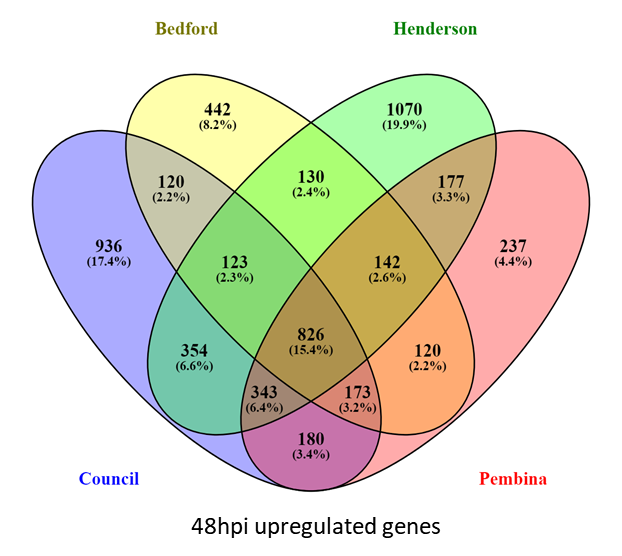 |
| 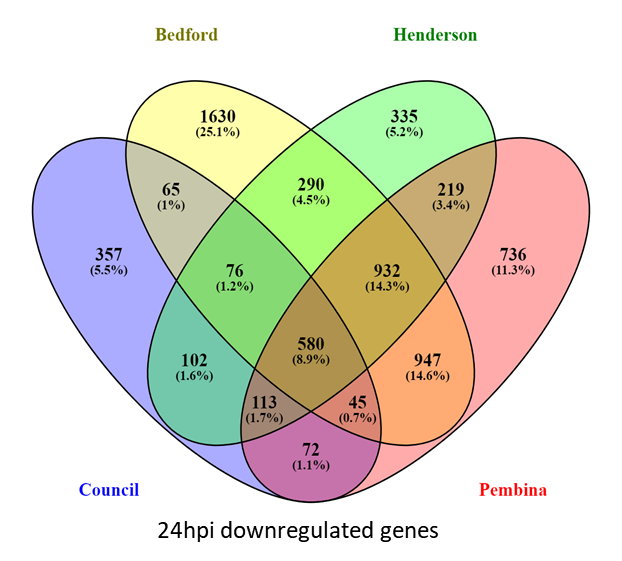 | 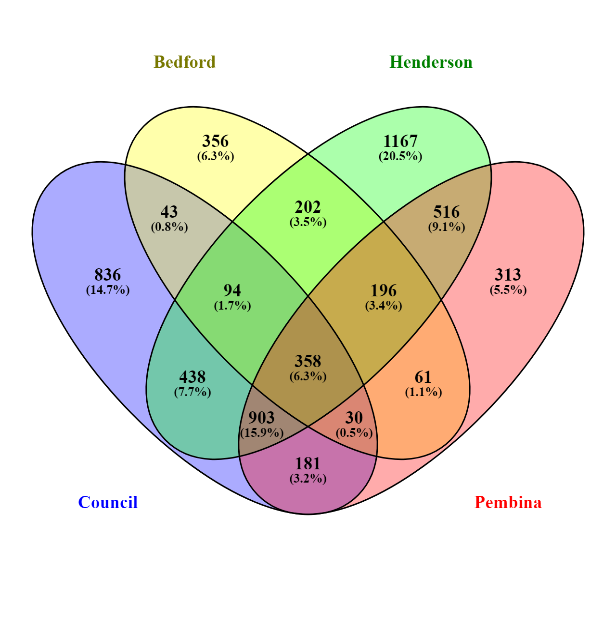 48hpi downregulated genes |

**Figure S1** Differntially expressed genes (DEGs) retrived from all four genotypes at 24 hpi and 48 hpi time of intervals compared to non-inoculated control. (A) Total numbers of DEGs (upregulated and downregulated) at each time points. (B) Venn diagram illustrating comparision of up and downregulated DEGs among all four genotypes, resistant (Bedford and Council) and susceptible (Henderson and Pembina) each time point.

B

| **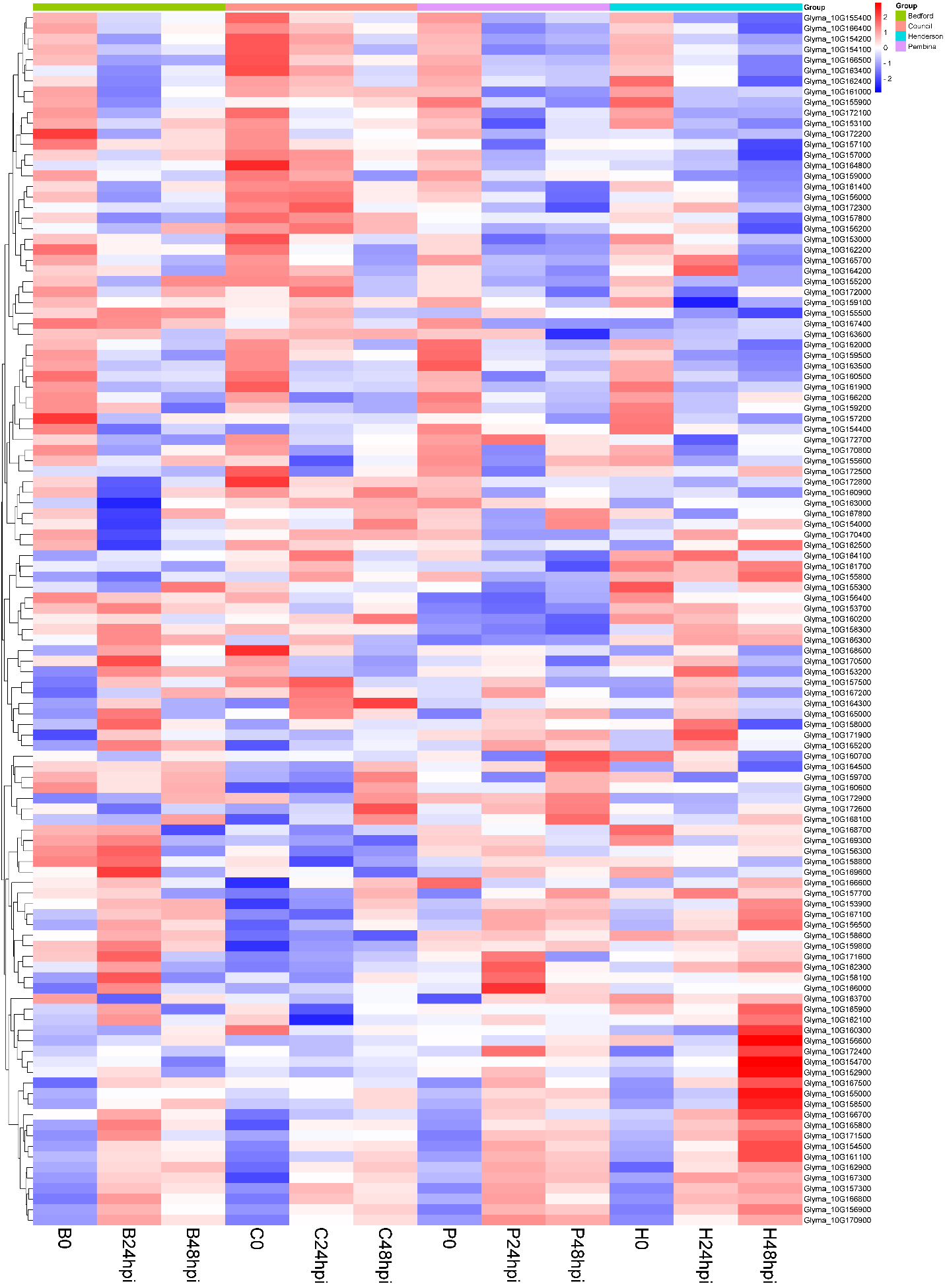**  A |
| --- |
| **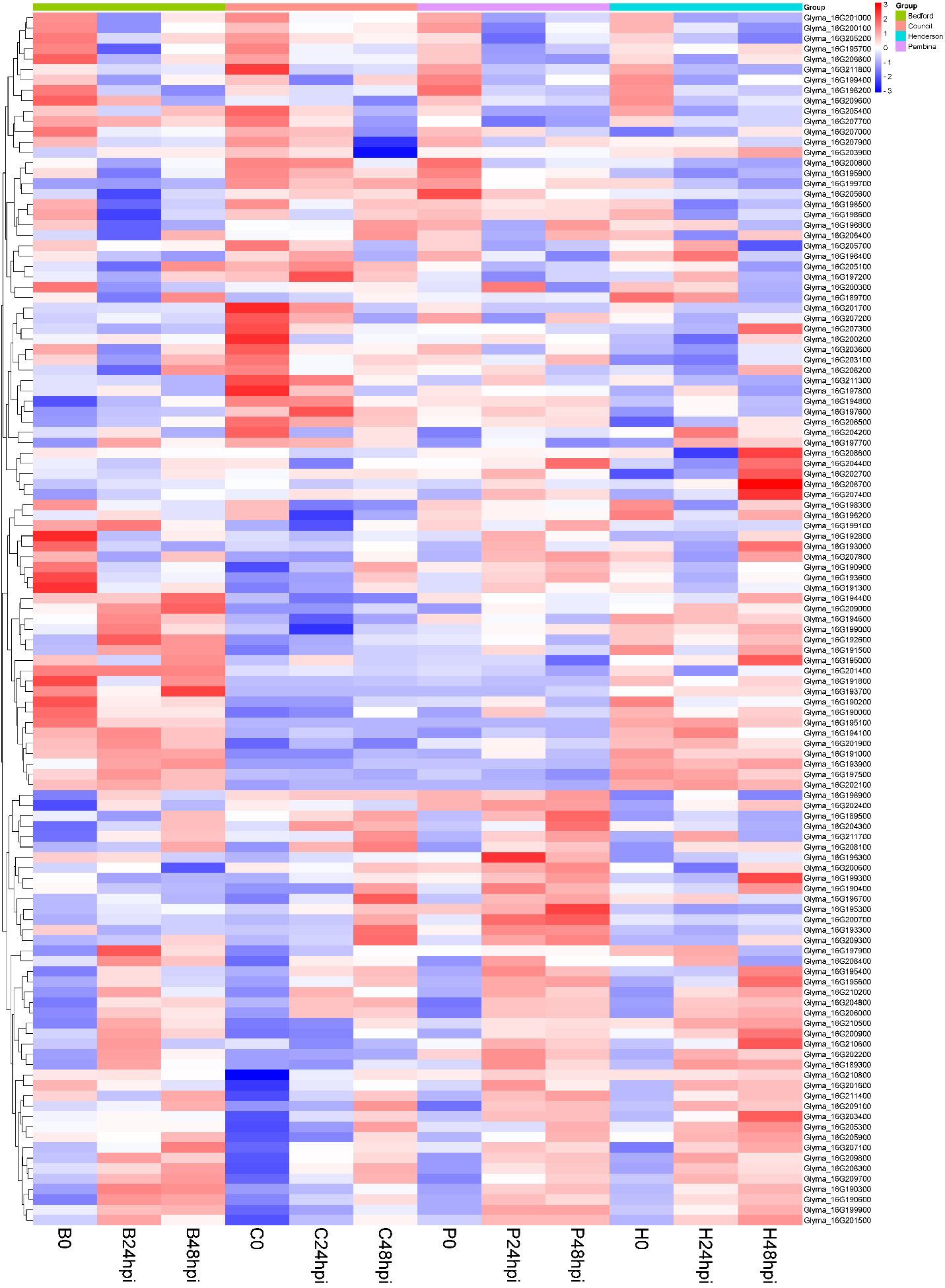**  B |

**Figure** and (B) Chromosome 16. These heat maps effectively captured the log2fold-change-based expression profiles of these genes when comparing different time points post-inoculation (24 hpi and 48 hpi) of *C. cassiicola* with control conditions across all four genotypes. Gene annotation information was sourced from Soybase to provide context to these genes.
